# Mechanisms of spikelet generation in cortical pyramidal neurons

**DOI:** 10.1186/1471-2202-16-S1-P121

**Published:** 2015-12-18

**Authors:** Martina Michalikova, Michiel Remme, Richard Kempter

**Affiliations:** 1 Institute for Theoretical Biology, Department of Biology, Humboldt-Universität zu Berlin, 10115 Berlin, Germany; 2 Bernstein Center for Computational Neuroscience, 10115 Berlin, Germany

## 

Spikelets are brief, spike-like depolarizations of small amplitude (< 20 mV) that can be measured in somatic intracellular recordings. Prominent spikelet activity was demonstrated in hippocampal CA1 pyramidal neurons in awake behaving [1,2] and anesthetized animals [3]. However, spikelets are rarely observed *in vitro*, and basic mechanisms underlying their generation in pyramidal neurons are not well understood.

Here we investigate the emergence of spikelets using mathematical analysis and numerical simulations of compartmental single-neuron models. Somatic spikelets are produced in the models upon orthodromic (somatic) stimulation. We find that spikelet occurrence depends on three main factors: A) Activation voltages of somatic channels need to be larger by several millivolts (~10 mV) than activation voltages of axonal sodium channels. B) The spike initiation zone (axon initial segment, AIS) has to be electrically segregated from the soma. C) The impedance mismatch between soma and AIS needs to be sufficiently large. In this way, weak orthodromic stimuli can trigger APs at the AIS that fail to activate somatic sodium channels and manifest as somatic spikelets. Stronger stimuli lead to full-size APs at the soma, either through axonal APs that backpropagate to the soma ('shouldered APs') or direct somatic AP generation ('full-blown APs').

Through analysis and simulations we isolated the cell parameters that allow for spikelet generation and identified possible causes of spikelet absence in *in vitro *preparations: First, the dendritic current sink *in vitro *is diminished due to "dendritic pruning" in slices. Next, the fraction of sodium channels usually available for (somatic) spiking is larger *in vitro *due to the overall lower spiking activity and lower resting membrane potential. Finally, the difference in activation voltages between somatic and axonal sodium channels under *in vitro *conditions might be smaller than under *in vivo *conditions as the activation voltage of sodium channels might be controlled by neuronal activity, which is typically much higher *in vivo *than *in vitro*.

## Conclusions

In our models, somatic spikelets represent APs that are only propagated down the axon, but are not backpropagated to the soma and the dendrites. Consequently, such a mechanism might be involved in the control of dendritic plasticity and/or in the homeostatic regulation of somato-dendritic firing rates without affecting the axonal output of a neuron.
